# Development of novel treatment strategies for inflammatory diseases—similarities and divergence between glucocorticoids and GILZ

**DOI:** 10.3389/fphar.2014.00169

**Published:** 2014-07-17

**Authors:** Qiang Cheng, Eric Morand, Yuan Hang Yang

**Affiliations:** Centre for Inflammatory Diseases, Department of Medicine, Southern Clinical School, Monash University Faculty of Medicine, Nursing and Health Sciences, Monash Medical CentreClayton, VIC, Australia

**Keywords:** glucocorticoids, GILZ, inflammation, anti-inflammation, immune response, cell biology

## Abstract

Glucocorticoids (GC) are the most commonly prescribed medications for patients with inflammatory diseases, despite their well-known adverse metabolic effects. Previously, it was understood that the anti-inflammatory effects of the GC/GC receptor (GR) complex were mediated via transrepression, whilst the adverse metabolic effects were mediated via transactivation. It has recently become clear that this “divergent actions” paradigm of GC actions is likely insufficient. It has been reported that the GC/GR-mediated transactivation also contributes to the anti-inflammatory actions of GC, via up-regulation of key anti-inflammatory proteins. One of these is glucocorticoid-induced leucine zipper (GILZ), which inhibits inflammatory responses in a number of important immune cell lineages *in vitro*, as well as in animal models of inflammatory diseases *in vivo*. This review aims to compare the GILZ and GC effects on specific cell lineages and animal models of inflammatory diseases. The fact that the actions of GILZ permit a GILZ-based gene therapy to lack GC-like adverse effects presents the potential for development of new strategies to treat patients with inflammatory diseases.

## Introduction

Glucocorticoids (GC) remain the most common prescription to treat patients with immune-mediated inflammatory diseases, despite their well-known side effects. The efficacy of GCs is underpinned by GC effects on intracellular pathways mediated via forming a complex with the glucocorticoid receptor (GR). The effects of the GC/GR complex depend on a combination of several effects. These include (i) transrepression, whereby the GC-GR complex tethers to pro-inflammatory transcription factors such as NF-κB and AP-1, constraining their activity; (ii) cis-repression, whereby the GC-GR complex binds directly to DNA and exerts inhibitory effects on gene transcription; and (iii) transactivation, whereby a GC-GR dimer acts as a *bona fides* transcription factor and activates gene transcription (McKay and Cidlowski, 1999; De Bosscher et al., 2003; Barnes, 2006; Beaulieu and Morand, 2011). Despite their beneficial effects, adverse effects of GC treatment have been noted since the beginning of their usage, because the amount of GCs required therapeutically to inhibit the immune system is in excess of metabolic homeostatic requirements. Until lately, it was believed that GC/GR mediated transrepression was predominantly responsible for the immune suppressive function of GCs, while GC/GR induced transactivation was linked to GC adverse metabolic effects (Reichardt et al., 2001; Schacke et al., 2005, 2007). Recently, however, a new role of GC/GR transactivation in immune suppressive actions has emerged, due to the functional characterization of several GC induced anti-inflammatory proteins. Many studies have shown that one such protein, glucocorticoid-induced leucine zipper (GILZ), inhibits activation of a wide range of immune cells under inflamed conditions (Beaulieu and Morand, 2011; Esposito et al., 2012; Cheng et al., 2013; Ngo et al., 2013a). Importantly, published data to date have not indicated GC-like adverse effects associated with GILZ, suggesting that GILZ exerts immunosuppressive effects that mimic those of GCs but occur via distinct pathways. In this review, we aim to summarize current knowledge of GILZ biological functions and compare them with the known effects of GC on immune system. The similarities and divergence between the effects of GILZ and GC suggest the potential use of GILZ-based therapies in improving GC efficacy while reducing GC metabolic toxicity, and thus the development of new treatment strategies to replace supplement or even replace GC.

## Glucocorticoids and glucocorticoid receptors

Inflammation is a self-protective process that forms part of the host organism responses to harmful insults, such as cell damage, foreign pathogens or irritants. To avoid excessive damage to the healthy tissues, the activated immune system needs to be rapidly “switched off” once the noxious stimuli are eliminated. Therefore, inflammatory responses are tightly regulated via competition between stimulative and suppressive signals. GC, produced by the adrenal gland, represents one of the most powerful endogenous pathways to temper the intensity of immune responses. Under certain conditions, however, the ability of endogenous GC to suppress immune response is overwhelmed, resulting in the hyper-activation of the immune system that leads to chronic auto-immune and inflammatory disease. In the majority of these diseases, treatment with synthetic GCs is used to control inflammation, exploiting the natural pathways that evolved to permit endogenous GC to regulate immune responses. The profound effectiveness of GCs underpins their use in a wide range of diseases. Despite their effectiveness, however, the use of GCs is accompanied by a litany of serious adverse effects, particularly in patients under long term or high dose treatment. These unwanted effects include diabetes, immunosuppression, osteoporosis and increased risk of cardiovascular events, all of which are closely associated with the physiological metabolic functions of GC (Rhen and Cidlowski, 2005). The continued use of glucocorticoids reflects a failure to discover a reliable GC replacement. To develop such glucocorticoid mimics, a large body of research has been conducted to understand the molecular actions of GC.

## Molecular mechanisms of glucocorticoid actions

**Figure 1:**
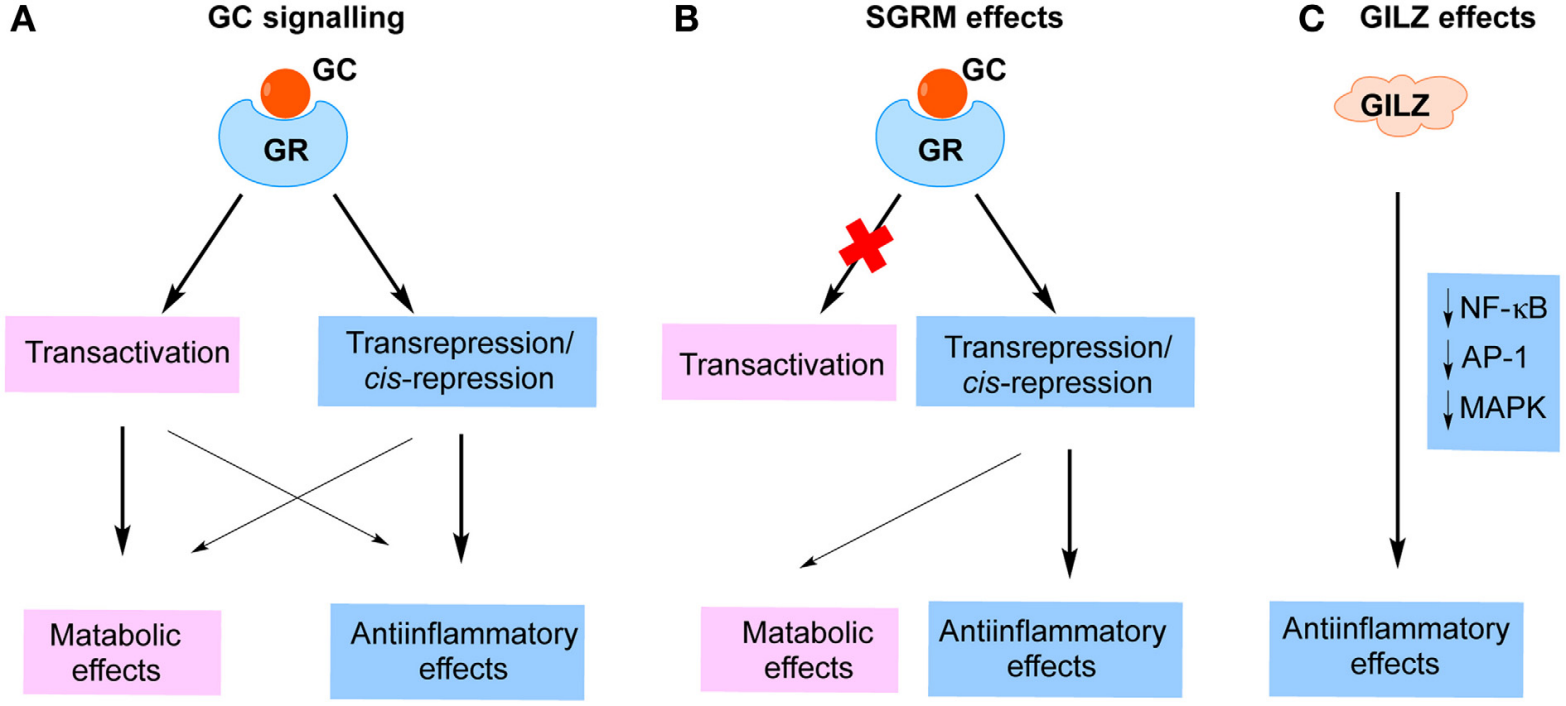
**Strategies to improve glucocorticoid therapy. (A)** Cellular effects of glucocorticoids (GC). GCs bind to glucocorticoid receptor (GR) and activate intracellular signaling pathways. Previously, it was believed that transactivation predominantly accounts for the adverse metabolic effects, whereas transrepression/cis-repression mainly mediates anti-inflammatory functions of GCs. **(B)** The strategy to develop “selective glucocorticoid receptor modulators (SGRM)” based on the paradigm that metabolic effects but not immune-suppressive effects depended on transactivation. **(C)** The anti-inflammatory effects of GILZ are independent of GR function and may conceivably avoid metabolic effects entirely.

GCs impact on intracellular pathways through binding to the GR (Rhen and Cidlowski, 2005). The GC/GR complex exerts its transrepression effects by tethering to and interfering with the function of proinflammatory transcription factors, such as NF-κB and AP-1, which consequently results in repression of a large number of proinflammatory mediators, cytokines, chemokines and adhesion molecules (Rhen and Cidlowski, 2005; Nixon et al., 2013). The GC/GR complex also suppresses gene expression via binding to negative GC response elements (nGRE) located within the promoters of genes, a phenomena called cis-repression (Newton, 2000; Clark and Belvisi, 2012; Nixon et al., 2013; Vandevyver et al., 2013). These GC/GR-induced transrepression and cis-repression effects are important in the therapeutic activity of GCs. On the other hand, the dimerized GC/GR complex is also capable of binding to positive GREs and thereby up-regulating the expression of multiple genes, many of which are related to the metabolic adverse effects of GCs (Rhen and Cidlowski, 2005; Clark and Belvisi, 2012; Nixon et al., 2013; Vandevyver et al., 2013). It has previously been believed that anti-inflammatory effects of GCs are mediated via transrepression, whereas the metabolic effects are mediated via transactivation (Figure 1A). Thus, a significant effort has been invested by pharmaceutical companies worldwide toward the discovery of “selective glucocorticoid receptor modulators” (SGRM), based on the idea that GR ligands that favor transrepression over transactivation would be equally effective but safer (Figure 1B) (Weinstein et al., 2011). As we have recently reviewed, however, it has become clear that the “divergent actions” paradigm of GC actions does not fully explain the parallel therapeutic and metabolic effects of GCs (Fan and Morand, 2012). For examples, nGREs were recently identified within the promoters of a large number of genes, including insulin, osteoprotegerin and anti-apoptotic proteins (Surjit et al., 2011). GC/GR binding to these nGREs results in down-regulation of gene expression, directly contributing to GC adverse metabolic effects. Moreover, GC/GR-dimer induced transactivation effects include the induction of the expression of several important anti-inflammatory proteins, including GILZ, annexin A1 (AnxA1) and MAPK phosphatase-1 (MKP-1) (Clark, 2003; Perretti and D'Acquisto, 2009; Beaulieu and Morand, 2011; Clark and Belvisi, 2012; Ratman et al., 2013; Yang et al., 2013). Indeed, two recent studies indicate that GC-GR-dimer-dependent effects, likely due to transactivation of anti-inflammatory proteins, are essential for the full anti-inflammatory effects of GCs (Baschant et al., 2011; Kleiman et al., 2012). These findings demonstrate a previously under-recognized role of GC-induced proteins in the anti-inflammatory effects of GC, suggesting that the paradigm separating GC anti-inflammatory and metabolic effects on the basis of transrepression and transactivation is inadequate to explain the effects of GC.

To develop a new strategy to mimic GC immune-suppressive functions but limit the metabolic effects, an alternate approach would be to discover GC-mediated inhibitory effects on the immune system that bypass the mechanism of GC metabolic effects, i.e., molecules that do not utilize the GR for their actions on inflammation. Accumulating evidence indicates that one of the GC-induced proteins, GILZ, represents the basis for such an approach (Figure 1C). GILZ has been detected and functionally characterized in a wide range of immune cells, including T lymphocytes, B lymphocytes, dendritic cells (DCs), monocyte/macrophages, mast cells and endothelial cells (ECs) (D'Adamio et al., 1997; Glynne et al., 2000; Berrebi et al., 2003; Cannarile et al., 2006; Cohen et al., 2006; Godot et al., 2006; Cheng et al., 2013). Within the most of cell types investigated, the main function of GILZ is immune suppressive. The molecular actions of GILZ were recently reviewed by us and others (Ayroldi and Riccardi, 2009; Beaulieu and Morand, 2011). Although GILZ expression is up-regulated by GCs, via GC/GR binding to GREs within the *Gilz* promoter (Asselin-Labat et al., 2005), divergent effects of GILZ and GCs on mesenchymal stem cell (MSC) differentiation have been reported, suggesting that GILZ lacks an ability to induce GC-related adverse effects (Shi et al., 2000; Zhang et al., 2008). This notion is further supported by the observation that GC immune suppressive functions do not always require the presence of GILZ, suggesting that GILZ and GCs work in parallel and impact on immune system through divergent signaling pathways (Cheng et al., 2013; Ngo et al., 2013a). In the following sections we shall review the known effects of GC and GILZ in various cells and compartments relevant to inflammatory disease and illustrate evidence that their effects are divergent.

## Comparison of the effects of GC and GILZ

To better understand the similarities and differences between GILZ and GCs, their effects on specific cell lineages and animal models of inflammatory diseases are directly compared in the following sections, as summarized in Tables 1, 2.

**Table 1:** **Comparison of GILZ and GC *in vitro* effects on specific cell lineages**.

	**GC effects**	**GILZ effects**
Thymocytes	↑ Apoptosis *via*	↑ Apoptosis *via*
	↓ Bcl-xL, NF-κB (Wang et al., 1999; Bianchini et al., 2006; Bruscoli et al., 2006)	↓ Bcl-xL, NF-κB (Delfino et al., 2004, 2006)
T lymphocytes	↓ Apoptosis *via*	↓ Apoptosis *via*
	↓ AP-1, FasL (Zacharchuk et al., 1990; Paliogianni et al., 1993; Yang et al., 1995)	↓ AP-1, FasL, FOXO3, BIM (Mittelstadt and Ashwell, 2001; Asselin-Labat et al., 2004; Latre de Late et al., 2010)
	↓ Th1 *via*	↓ Th1 *via*
	↓ IL-2, IFNγ (Daynes and Araneo, 1989; Miyaura and Iwata, 2002)	↓ IL-2, IFNγ (Ayroldi et al., 2001; Mittelstadt and Ashwell, 2001; Cannarile et al., 2006; Ngo et al., 2013a)
	↑ Th2 *via*	↑ Th2 *via*
	↑ IL-4, IL-10 (Daynes and Araneo, 1989; Miyaura and Iwata, 2002)	↑ GATA-3, STAT6, IL-4, IL-10 (Cannarile et al., 2006)
	↓ Activation *via*	↓ Activation *via*
	↓ AP-1, NF-κB, No effect on ERK (Vacca et al., 1992; Adcock et al., 1995; Tsitoura and Rothman, 2004)	↓ AP-1, NF-κB, NFAT, ↓ ERK (Ayroldi et al., 2001, 2002; Cannarile et al., 2006)
	No effect on Th17 (McKinley et al., 2008; Nanzer et al., 2013)	↓ Th17 *via*
		↓ IL17A (Ngo et al., 2013a)
Dendritic cells	↓ Maturation *via*	↓ Maturation *via*
	↓ CD80, CD86, IL-12 (Kitajima et al., 1996; Rea et al., 2000)	↓ CD80, CD86 (Cohen et al., 2006)
	↑ Tolerance *via*	↑ Tolerance *via*
	↑ IL-10, B7-H1 (Rea et al., 2000; Cohen et al., 2006)	↑ IL-10, B7-H1 (Cohen et al., 2006)
	↑ DC induced Treg *via*	↑ DC induced Treg *via*
	↑ FOXP3+ Treg (Hamdi et al., 2007b; Unger et al., 2009)	↑ FOXP3+ Treg (Hamdi et al., 2007b)
Endothelial cells	↓ Activation *via*	↓ Activation *via*
	↓ NF-κB, p38, ↑ MKP-1 (Ray et al., 1997; Furst et al., 2007; Cheng et al., 2013)	↓ NF-κB, p38, ERK, JNK, ↑ MKP-1 (Cheng et al., 2013)
Monocyte/Macrophages	↓ Activation *via*	↓ Activation *via*
	↓ NF-κB, IL-1β, TNFα (Jeon et al., 2000; Steer et al., 2000; Joyce et al., 2001)	↓ NF-κB, IL-1β, TNFα (Berrebi et al., 2003; Hamdi et al., 2007a)
MSC	↑ AP, ↓ OB *via*	↓ AP, ↓ OB *via*
	↑ C/EBPδ, PPARγ2 (Shi et al., 2000; Yao et al., 2008)	↓ PPARγ2 (Shi et al., 2003; Zhang et al., 2008)

The effects of GCs and GILZ on each cell lineage and related mechanisms are shown. Underlined text indicates the GILZ and GC effects are divergent. Abbreviation: AP, adipocytes; OB, osteoblasts; MSC, mesenchymal stem cells.

**Table 2:** **Comparison of GILZ and GC *in vivo* effects on animal models of diseases**.

**Diseases**	**GC effects**	**GILZ effects**	**Techniques**
Th1 Colitis (DNBS)	↓ *via*	↓ *via*	GILZ TG
	↓ TNFα, IL-6 (Antonioli et al., 2007)	↓ NF-κB, TNFα, IFNγ, FasL (Cannarile et al., 2009)	
Th2 Colitis (Oxazolone)	Unknown	↑ *via*	
		↑ MPO (Cannarile et al., 2009)	
SCI	↓ *via*	↓ *via*	
	↓ ROS (Hall, 2011)	↓ NF-κB, TNFα, FasL, ↓ Bcl-2 (Esposito et al., 2012)	
EAE	↓ (Chen et al., 2006)	↓ *via*	rGILZ
		↓ NF-κB, IFNγ, IL-17, ↓ GATA-3 (Srinivasan and Janardhanam, 2011)	
Experimental arthritis	↓ CIA, AIA, K/BxN (Yang et al., 2004; Beaulieu et al., 2010; Patel et al., 2012; Ngo et al., 2013a)	↓ CIA *via*	GILZ-AAV
		↓ IL-1, TNFα (Beaulieu et al., 2010; Ngo et al., 2013a)	
		No effect (Ngo et al., 2013a)	Endogenous GILZ via KO
Endotoxemia	↓ (Yang et al., 2009)	↓ *via*	SPRET/Ei mice
		↓ IL-6 (Pinheiro et al., 2013)	
		No effect(Ngo et al., 2013a)	Endogenous GILZ via KO
DTH	↓ (Taube and Carlsten, 2000)	↓ *via*	Endogenous GILZ via KO
		↓ IFNγ, IL-17, proliferation (Ngo et al., 2013a)	
Male infertility	↑ SSC apoptosis *via*	↓ SSC apoptosis *via*	
	↑ FasL (Khorsandi et al., 2008; Orazizadeh et al., 2010)	↓ ERK, AKT, FOXO1, BIM (Bruscoli et al., 2012; Ngo et al., 2013b)	

The effects of GCs and GILZ on each disease model and related mechanisms are shown. Underlined text indicates the GILZ and GC effects are divergent. The technique by which GILZ function was examined is also listed. MPO, myeloperoxidase; SCI, spinal cord injury; EAE, experimental autoimmune encephalomyelitis; CIA, collagen-induced arthritis; AIA, antigen-induced arthritis; SPRET/Ei, a wild-derived inbred murine strain; SSC, spermatogonia stem cells; DTH, delayed-type hypersensitivity.

## Thymocytes

GCs induce thymocyte apoptosis and this effect requires GR-dependent protein synthesis (Thomas et al., 1983; Cohen and Duke, 1984; Ashwell et al., 2000; Bruscoli et al., 2006). Although the mechanisms remain only partially understood, mitochondrial apoptotic signaling pathways mediated by Bcl-2 family members play a dominant role (Sentman et al., 1991; Grillot et al., 1995). In addition, GCs suppress NF-κB signals in thymocytes, leading to increased thymocyte cell death (Wang et al., 1999). GILZ was initially discovered in murine thymocytes as its expression is exquisitely sensitive to GCs (D'Adamio et al., 1997). To elucidate the role of GILZ in regulation of thymocyte survival, a transgenic (TG) mouse line, in which GILZ expression is driven by a CD2 promoter, was generated (Delfino et al., 2004). Consistent with GC pro-apoptotic effects, overexpression of GILZ led to a reduction of Bcl-xL expression and accelerated anti-CD3 Ab induced thymocyte apoptosis, which was accompanied by inhibition of NF-κB p65 nuclear translocation and DNA binding ability (Delfino et al., 2004, 2006). As a result, adult TG mice show a decrease in CD4 and CD8 double positive thymocytes. Moreover, a mouse strain (*GR^lck−Cre^*), in which GR expression is conditionally knocked out in thymocytes prior to selections, was recently generated (Mittelstadt et al., 2012). Deletion of GR led to an absence of GC signaling and GC-induced GILZ expression. As a result, *GR^lck−Cre^* thymoctyes are completely resistance to GC-induced apoptosis. Thus, these studies suggest that GCs and GILZ both play a similar pro-apoptotic role in regulation of thymocyte apoptosis.

## T lymphocytes

### Activation-induced apoptosis

In contrast to their pro-apoptotic effects on thymocytes, GC and GILZ effects on activated T lymphocytes are anti-apoptotic (D'Adamio et al., 1997). GCs directly interfere with AP-1 and suppress activation-induced FasL expression, to inhibit T lymphocyte apoptosis (Paliogianni et al., 1993; Yang et al., 1995). The GC dexamethasone (DEX) also blocks NF-κB activation through inhibiting IκB degradation, to reduce anti-CD3-induced FasL expression and T cell death (Auphan et al., 1995; Liberman et al., 2012). Similarly, GILZ inhibits anti-CD3 Ab-induced cell apoptosis in a T cell line via directly interfering with the AP-1 transcription factor, leading to inhibition of FasL expression and pro-apoptotic signaling (D'Adamio et al., 1997; Mittelstadt and Ashwell, 2001). GILZ overexpression also protects IL-2 withdrawal-induced T lymphocyte cell death, which is correspondingly accelerated in GILZ-deficient cells (Asselin-Labat et al., 2004). IL-2 withdrawal rapidly induces binding of the FOXO3 transcription factor to the GILZ promoter, leading to increased GILZ expression (Asselin-Labat et al., 2004, 2005). On the other hand, GILZ inhibits FOXO3 transcriptional activity via CRM1 (a nuclear transport receptor)-dependent nuclear exclusion of FOXO3 (Latre de Late et al., 2010), leading to decreased expression of itself and BIM, a pro-apoptotic member of the Bcl-2 family. These findings suggest a bidirectional regulation between FOXO3 and GILZ proteins to regulate cell apoptosis in T lymphocytes.

### Immune response

GCs suppress Th1 development and promote Th2 differentiation in CD4 T lymphocytes, through mediating the production of a wide range of cytokines at the transcriptional level (Kunicka et al., 1993; Almawi et al., 1996; Miyaura and Iwata, 2002). This notion is supported by the observation that GCs inhibit the activation of several important T cell pro-inflammatory transcription factors, including AP-1, NFAT and NF-κB (Tsitoura and Rothman, 2004). Upon TCR activation of murine CD4 T cells, GCs reduce production of Th1 cytokines (e.g., IL-2) and promote the expression of Th2 cytokines (e.g., IL-4) (Daynes and Araneo, 1989). It is now clear that GCs suppress IL-2 expression via direct inhibition of AP-1 and NF-κB DNA binding activity (Vacca et al., 1992; Adcock et al., 1995). A similar skew toward Th2 phenotype was observed in T lymphocytes from GILZ TG mice (Cannarile et al., 2006). Anti-CD3/anti-CD28 antibody-induced activation of the Th2 specific transcription factors GATA3 and STAT6 is increased in GILZ TG T lymphocytes, whereas activity of T-bet, a Th1 transcription factor, was reduced (Cannarile et al., 2006). As a result, GILZ induces T lymphocytes to produce more Th2 cytokines (e.g., IL-4, IL-5, and IL-10) and less Th1 cytokines (e.g., INF-γ). The concept that GILZ suppresses Th1 responses is supported by the observation that increased IFN-γ production is detected in GILZ knockout (KO) T cells (Ngo et al., 2013a). Moreover, GILZ inhibits the transcriptional activity of AP-1, NF-κB and NFAT, transcription factors known to regulate T cell activation and differentiation (Ayroldi et al., 2001, 2002; Mittelstadt and Ashwell, 2001), thus mimicking GC effects. GILZ directly interacts with NF-κB p65 subunit through its C-terminal domain (Di Marco et al., 2007), and inhibits p65 nuclear translocation and DNA binding, resulting in reduced expression of IL-2 and IL-2R (Ayroldi et al., 2001). On the other hand, the N-terminal of GILZ interacts with AP-1 and subsequently inhibits AP-1 DNA binding, resulting in decreased IL-2 expression (Mittelstadt and Ashwell, 2001).

Despite many similarities, several divergent effects of GCs and GILZ on T lymphocytes activation and differentiation have also been reported. GC effects on Th17 phenotype are not fully understood. For example, GCs have no effect on Th17 cytokine production from polarized Th17 T cells *in vitro* (McKinley et al., 2008). As a result, neutrophil infiltration induced by adoptive transfer of Th17 cells cannot be attenuated by GCs *in vivo*. Consequently, the increased production of IL-17A and IL-22 by asthmatic patient peripheral blood monocytes (PBMC) is insensitive to GCs *in vitro* (Nanzer et al., 2013). In contrast, early evidence suggests that GILZ inhibits IL-17A production as GILZ KO T cells produce significantly more IL-17A than WT cells (Ngo et al., 2013a). Moreover, GCs have no effect on phosphorylation of ERK MAP kinase in T lymphocytes activated by anti-CD3 Ab (Tsitoura and Rothman, 2004), whereas GILZ markedly inhibits ERK activation (Ayroldi et al., 2002). GILZ directly interacts with and inhibits Ras/Raf, upstream kinases in the ERK pathway (Ayroldi et al., 2002, 2007; Soundararajan et al., 2007). Together, these observations indicate that while GILZ mimics most GC effects on T cell activation, effects on IL-17A production and ERK activation are divergent.

## Dendritic cells

DCs play a crucial role in priming naive T lymphocytes via their ability to recognize and present antigens to T cells (Hackstein and Thomson, 2004). GCs and GILZ have very similar suppressive effects on DC activation, and many GC effects have been suggested to be GILZ-dependent (Cohen et al., 2006; Hamdi et al., 2007b). To mature, immature DCs need to be activated by pro-inflammatory stimuli (e.g., TNFα), bacterial components (e.g., LPS) or T lymphocytes expressing CD40 ligand (CD40L) (Hackstein and Thomson, 2004). GCs inhibit DC maturation via suppressing expression of DC maturation markers (e.g., CD80, CD86, CD83 and IL-12), and promoting tolerance markers (e.g., IL-10 and B7-H1) (Kitajima et al., 1996; Rea et al., 2000; Cohen et al., 2006). GCs also inhibit chemokine production (e.g., CCL-3, CCL-5 and CXCL-8) by human DCs and, therefore, impair DC ability to activate T cells (Cohen et al., 2006). Moreover, GC-treated DCs promote the generation of regulatory T (Treg) cells derived from pathogen-specific human CD4 T lymphocytes (Hamdi et al., 2007b). Hamdi et al. reported that GC treatment increases DC ability to induce the CD25^hi^FOXP3 phenotype among human CD4^+^ T cells. The function of these inducible Tregs is confirmed by their capacity to inhibit human PBMC proliferation (Hamdi et al., 2007b). GILZ expression is highly sensitive to GC treatment in human DCs and GILZ overexpression mimics the known GC inhibitory effects on DC maturation and activation (Cohen et al., 2006; Hamdi et al., 2007b). Consistent with this, deficiency of endogenous GILZ induced by silencing via siRNA reverses these GC effects, suggesting that GILZ expression plays a critical role in mediating GC actions in DCs (Cohen et al., 2006).

## Endothelial cells

ECs play a critical role in mediating the recruitment of fast-traveling leukocytes from the blood stream to inflamed tissues. This occurs via upregulation of the pro-inflammatory adhesion molecules (E-selectin, VCAM-1 and ICAM-1), and cytokines and chemokines such as IL-6, CXCL8 and CCL2 (Ley et al., 2007). The expression of endothelial pro-inflammatory molecules is predominantly controlled by NF-κB and MAP kinase signaling pathways (Chen and Manning, 1995; Kuldo et al., 2005; Cheng et al., 2010). GC effects on the activation of human umbilical cord vein endothelial cells (HUVECs) require GR, as GR-deficient HUVECs show prolonged NF-κB activation (Goodwin et al., 2013). GCs inhibit E-selectin, IL-6, VCAM-1 and ICAM-1 expression in HUVECs via interference with NF-κB (Ray et al., 1997; Pan et al., 2011; Goodwin et al., 2013). Additionally, GCs regulate endothelial activation via NF-κB-independent mechanisms (Furst et al., 2007). In HUVEC, GCs induce the expression of MKP-1, a MAPK inhibitory phosphatase, leading to inhibition of p38 MAPK-dependent E-selectin expression without affecting NF-κB DNA binding activity (Furst et al., 2007). Consistent with these observations, GC-treated HUVECs have impaired ability to support leukocyte rolling and transmigration under flow conditions (Cheng et al., 2013). GILZ is highly expressed in ECs in synovial tissue from patients with rheumatoid arthritis (RA), and plays an important role in regulation of endothelial adhesive functions (Beaulieu et al., 2010; Cheng et al., 2013). Similar to the known GC effects, GILZ expression in HUVECs inhibits their capacity to support leukocyte rolling, adhesion and transmigration, accompanied by reduced expression of E-selecting, ICAM-1, IL-6, CXCL8, and CCL2 (Cheng et al., 2013). Differing from observations reported in T lymphocytes, however, in a human microvascular endothelial cell line (HMEC), GILZ inhibits NF-κB transcription activity via suppressing NF-κB p65 DNA binding ability, without affecting its nuclear translocation, suggesting a novel mechanism by which GILZ regulates NF-κB signaling in human ECs. GILZ also upregulates MKP-1 expression and, thereby, inhibits TNFα-induced p38, ERK, and JNK MAP kinase activation, mimicking GC effects. On the other hand, GILZ silencing via siRNA does not alter the inhibitory effects of exogenous GC on HUVEC adhesive function, suggesting redundancy of GILZ in GC effects on ECs despite the GC-mimicking effects of GILZ (Cheng et al., 2013). Together, these observations suggest that GCs and GILZ both play an anti-inflammatory role in regulation of human EC activation, although GILZ is not required for GC actions.

## Monocytes/macrophages

GCs inhibit monocyte/macrophage activation via suppressing AP-1 and NF-κB signaling, leading to reduced expression of a wide range of pro-inflammatory genes (Mukaida et al., 1991; Berkman et al., 1995; Marfaing-Koka et al., 1996; Jeon et al., 2000; Steer et al., 2000; Cao et al., 2003). GILZ is constitutively expressed in monocytes/macrophages and its expression is also sensitive to GC treatment (Berrebi et al., 2003; Hamdi et al., 2007a). GILZ inhibits LPS-induced production of cytokines and chemokines such as TNFα, RANTES, IL-6, and IL-1β by human monocytes and macrophages *in vitro*, mimicking the effects of GC (Hamdi et al., 2007a; Wang et al., 2012). Mechanistic studies in THP-1 cells, a human monocyte cell line, show that GILZ directly interacts with the NF-κB p65 subunit and suppresses transcriptional activity, resulting in reduced expression of macrophage activation markers CD80, CD86, and TLR2, and chemoknies CCL5 and CCL3 (Berrebi et al., 2003). Moreover, another GC induced anti-inflammatory protein, AnxA1, requires GILZ to mediate inhibitory effects of GC in murine macrophage (Yang et al., 2009). In keeping with these findings, GILZ expression is inhibited in macrophages from patients with Crohn's disease, tuberculosis and alcoholic hepatitis (AH), typical inflammatory diseases associated with macrophage activation (Berrebi et al., 2003; Hamdi et al., 2007a). This impairment of GILZ expression has been suggested to be implicated in prolonged macrophage responses in these patients. GILZ mRNA stability was found to be reduced in human macrophages upon Toll-like receptor (TLR) activation (Hoppstadter et al., 2012), suggesting a mechanism for permissive reductions in GILZ expression during inflammation. LPS triggers TLR signaling and caused GILZ mRNA degradation in a tristetraprolin (TTP) and MyD88 dependent manner (Hoppstadter et al., 2012). Together, these observations support the notion that GILZ and GCs have similar inhibitory effects on monocyte/macrophages activation via interference with NF-κB signaling.

## Mesenchymal stem cells (MSCs)/osteoblasts

One of the main functions of multipotent MSCs is to differentiate into important cell lineages, including adipocytes and osteoblasts (Pittenger et al., 1999). The differentiation of MSCs into adipocytes and osteoblasts is reciprocally modulated, mainly via regulation of the activity of peroxisome proliferator-activated receptor gamma (PPARγ2), a master transcription factor that promotes adipogenic differentiation (Tontonoz et al., 1994; Weinstein et al., 1998; Ahdjoudj et al., 2001; Akune et al., 2004). GCs induce MSC differentiation toward adipocytes and suppress osteogenic differentiation, via promoting CCAAT/enhancer-binding protein δ (C/EBPδ) expression and its binding to the C/EBP binding site within the PPARγ2 promoter, resulting in increased PPARγ2 mRNA expression and transcriptional activity (Shi et al., 2000; Yao et al., 2008). This GC inhibitory effect on osteoblast formation accounts in part for the rapid bone loss observed in patients during GC treatment. Despite the fact that GCs rapidly induces GILZ expression in MSCs, GILZ exerts a completely opposite effect in the regulation of MSC differentiation (Eddleston et al., 2007). GILZ expression in MSCs increases osteogenic differentiation, and inhibits adipocyte formation (Shi et al., 2003; Zhang et al., 2008). GILZ was reported in these studies to bind to the C/EBP site in competition with C/EBPδ, leading to a decreased PPARγ2 expression. Together, these observations suggest that GC and GILZ play opposite roles in regulation of adipocyte and osteoblast formation.

## Animal models of inflammatory diseases

To better understand the role of GILZ in the regulation of inflammation, GILZ effects on animal models of inflammatory diseases have been studied. Consistent with GILZ effects on Th1/Th2 balance *in vitro*, the GILZ TG mice showed increased sensitivity to bleomycin-induced neutrophil infiltration and edema, a typical Th2 dependent model of inflammation (Cannarile et al., 2006). Similarly, GILZ TG mice were more susceptible to oxazolone induced Th2 colitis (Cannarile et al., 2009), consistent with the pro-Th2 and anti-Th1 effects of GILZ discussed above. On the other hand, a beneficial effect of GILZ has been described in several animal models of Th1-related inflammatory diseases. T cell infiltration is reduced in GILZ TG mice during dinitrobenzene sulfonic acid (DNBS)-induced colitis, accompanied by a decrease in tissue damage, epithelial cell apoptosis, cytokine production, as well as NF-κB activation (Cannarile et al., 2009). Using the same GILZ TG mice, similar protective effects of GILZ were also detected in a spinal cord injury (SCI) model, mainly due to the GILZ inhibition of T cell activation and recruitment (Esposito et al., 2012). Consistently, an ability of GILZ to suppress inflammation was demonstrated in a rat model of experimental autoimmune encephalomyelitis (EAE) using an amphipathic chariot peptide (Pep-1) for systemic administration of GILZ proteins or peptides (Srinivasan and Janardhanam, 2011). Recently, the role of GILZ in the murine collagen-induced arthritis (CIA) model of RA was investigated, via local induction of GILZ expression in response to local injection of GILZ-adeno-associated virus (GILZ-AAV) into the joints (Ngo et al., 2013a). GILZ expression inhibited disease development in the CIA model, mimicking the effects of therapeutic dosing with the GC dexamethasone. Consistent with this observation, GILZ depletion by systemic administration of GILZ siRNA was previously found to increase disease severity in CIA (Beaulieu et al., 2010). Moreover, the anti-inflammatory function of GILZ has been demonstrated in a wild-derived inbred SPRET/Ei mouse strain (Pinheiro et al., 2013). These mice are resistant to LPS-induced endotoxemia, as a result of increased GILZ expression due to genetic variations (Pinheiro et al., 2013). LPS-induced IL-6 and IL-12 production were also reduced in SPRET/Ei macrophages, and silencing GILZ by siRNA, in contrast, completely abolished this effect (Pinheiro et al., 2013). These studies provide direct evidence that GILZ is a key mediator of inflammatory responses in both innate and adaptive immune systems.

To further study biological functions of endogenous GILZ, GILZ knockout (KO) mouse strains were recently generated independently by four laboratories (Bruscoli et al., 2012; Romero et al., 2012; Suarez et al., 2012; Ngo et al., 2013a). Surprisingly, male KO animals are infertile and lack the ability to produce sperm. Further research demonstrated that GILZ plays critical role in regulation of spermatogonia stem cell (SSC) survival and differentiation, likely via mediating ERK, AKT, and FOXO1 activity (Bruscoli et al., 2012; Ngo et al., 2013b). On the other hand, no major alteration in immune responses was detected in GILZ KO mice, despite a moderate increase in T cell proliferation and DTH response (Ngo et al., 2013a). Surprisingly, given the effects of GILZ therapy, GILZ deficiency failed to alter disease phenotypes in antigen-induced arthritis (AIA), K/BxN serum-transfer arthritis, CIA, and LPS-induced cytokinemia (Ngo et al., 2013a). GILZ deficiency also had no effect on GC sensitivity in these models (Ngo et al., 2013a). Consistent with this, bone marrow macrophages of GILZ KO mice showed neither impaired inflammatory response to LPS, nor reduced level of sensitivity to GCs, suggesting that GILZ is redundant for GC immune suppressive functions in these phenomena (Suarez et al., 2012). This result is in line with the findings described in ECs, in which GC actions were not GILZ dependent despite inhibitory actions of GILZ in these cells (Cheng et al., 2013). In contrast, GC effects on DCs were abrogated on GILZ silencing, suggesting variation in GILZ and GC impacts in different cell types. Further research is required to elucidate the divergent molecular pathways involved in GILZ and GC actions in these cells.

## Conclusions

The studies reviewed here suggest that GILZ and GCs share many anti-inflammatory effects on immune cells. However, differences between GILZ and GC impacts on immune cell function and animal models of disease are evident, indicating that the mechanisms involved in the actions of GILZ and GC in specific cell types may be different. Moreover, the requirement for GILZ in GCs actions varies between different cell types, suggesting that GILZ mediation of GCs functions is cell-type-specific. The opposite effects of GILZ and GCs on MSC differentiation raise the possibility that development of a GILZ based gene therapy to treat inflammatory disease could result in effects opposite to those of GC in terms of adiposity and osteoporosis. Further investigation is required to determine whether GILZ induces GC-related adverse metabolic effects. If confirmed, this will support the hypothesis that GILZ therapeutic effects mimic those of GCs but lack GC-like metabolic effects. As a result, GILZ-based gene therapy has great potential in the therapy of human diseases.

## Key concepts

1. GILZ inhibits inflammatory responses in a number of important immune cell lineages *in vitro*;
2. GILZ reduces disease severity in animal models of inflammatory diseases *in vivo*, mimicking glucocorticoid effects;
3. GILZ exerts inhibitory effects on immune system independent of glucocorticoid receptor and, thus, avoids glucocorticoid-induced adverse metabolic effects;
4. Further investigation is required to confirm the lack of ability of GILZ to induce GC-related side effects;
5. GILZ-based gene therapy has great potential in development of new treatment strategies for human diseases.

### Conflict of interest statement

The authors declare that the research was conducted in the absence of any commercial or financial relationships that could be construed as a potential conflict of interest.
